# The neurogenic phase of angiotensin II–salt hypertension is prevented by chronic intracerebroventricular administration of benzamil

**DOI:** 10.1002/phy2.245

**Published:** 2014-02-26

**Authors:** John W. Osborn, Dalay M. Olson, Pilar Guzman, Glenn M. Toney, Gregory D. Fink

**Affiliations:** 1 Department of Integrative Biology and Physiology, University of Minnesota, Minneapolis, Minnesota; 2 Department of Physiology and Center for Biomedical Neuroscience, University of Texas Health Science Center at San Antonio, San Antonio, Texas; 3 Department of Pharmacology and Toxicology, Michigan State University, East Lansing, Michigan

**Keywords:** Neurogenic hypertension, sympathetic nerve activity, salt‐dependent hypertension

## Abstract

Hypertension induced by chronic administration of angiotensin II (AngII) is exacerbated by high‐salt intake. Previous studies have demonstrated that this salt‐sensitive component is due to increased activity of the sympathetic nervous system, suggesting an interaction of plasma AngII with sodium‐sensitive regions of the brain. This study tested the hypothesis that the salt‐sensitive component of AngII‐induced hypertension would be prevented by intracerebroventricular (ICV) administration of the sodium channel/transporter blocker benzamil. Male Sprague Dawley rats were instrumented to measure mean arterial pressure (MAP) by radio telemetry and for ICV administration of benzamil or vehicle and placed in metabolic cages for measurement of sodium and water intake and excretion. In rats consuming a high‐salt diet (2.0% NaCl) and treated with ICV vehicle, administration of AngII (150 ng/kg/min, sc) for 13 days increased MAP by ~30 mmHg. ICV administration of benzamil (16 nmol/day) had no effect during the first 5 days of AngII, but returned MAP to control levels by Day 13. There were minimal or no differences between ICV vehicle or benzamil groups in regards to sodium and water balance. A lower dose of ICV benzamil administered ICV at 8 nmol/day had no effect on the MAP response to AngII in rats on a high‐salt diet. Finally, in contrast to rats on a high‐salt diet, AngII had negligible effects on MAP in rats consuming a low‐salt diet (0.1% NaCl) and there were no differences in any variable between ICV benzamil (16 nmol/day) and ICV vehicle‐treated groups. We conclude that the salt‐sensitive component of AngII‐induced hypertension is dependent on benzamil blockable sodium channels or transporters in the brain.

## Abstract (precis)

Chronic intracerebroventricular infusion of benzamil at 16 nmol/day attenuates AngII–salt hypertension. This effect is not observed at a dose of 8 nmol/day.

## Introduction

It has long been known that the severity of angiotensin II (AngII)‐induced hypertension in experimental animals is directly dependent on the prevailing level of salt intake (Cowley and McCaa 1976). Recent studies suggest that this salt sensitivity results from activation of central neural mechanisms that increase sympathetic nerve activity (SNA; Osborn et al. 2011). Specifically, our group has reported that the magnitude of the depressor response to ganglionic blockade becomes progressively greater during chronic AngII administration to rats consuming a high‐salt diet (i.e., AngII‐salt), but not a normal or low‐salt diet (King et al. 2007; Kuroki et al. 2012). Similarly, another indirect measure of “whole body” sympathetic activity, whole‐body norepinephrine spillover, is increased by treatment with AngII of rats on a high‐salt diet, whereas this sympathoexcitatory response is not observed in rats on a normal‐salt diet (King and Fink 2008). Activation of SNA in AngII–salt rats is organ specific and delayed in onset. Specifically, sympathetic activity in AngII–salt rats targets the splanchnic vascular bed and is not evident until several days after the start of AngII administration (King et al. 2007; Yoshimoto et al. 2010; Osborn et al. 2011; Kuroki et al. 2012).

The observation that the neurogenic component of AngII‐induced hypertension is exacerbated by a high‐salt diet that itself does not increase arterial pressure suggests a synergistic interaction between circulating AngII and sodium‐sensitive neural mechanisms that regulate SNA (Osborn and Fink 2010). Although mechanisms of this interaction between circulating AngII and sodium‐sensitive regions of brain are not well understood, an important clue is that “salt‐sensitive neurogenic hypertension” in other animal models such as Dahl salt‐sensitive rats (Gomez‐Sanchez and Gomez‐Sanchez 1995; Wang and Leenen 2002), stroke‐prone spontaneously hypertensive rats (Nishimura et al. 1998) and DOCA‐salt‐treated rats (Nishimura et al. 1998; Abrams et al. 2010) is prevented and/or reversed by intracerebroventricular (ICV) administration of amiloride, or its analogue benzamil. Which specific amiloride‐sensitive proteins in the brain mediate these antihypertensive effects is currently unknown, but epithelial sodium channels (ENaC) or a central nervous system homolog have been proposed as likely candidates (Abrams and Osborn 2008). ENaC, which mediate aldosterone‐stimulated sodium transport in the distal nephron, are also modulated by circulating AngII (Peti‐Peterdi et al. 2002). Collectively, these observations have led to the concept that aldosterone and AngII might also modulate channels expressed in brain that resemble ENaC to increase SNA and thereby induce salt‐sensitive neurogenic models of hypertension (Huang et al. 2006).

This study was designed to test the hypothesis that the salt‐sensitive component of AngII‐induced hypertension, similar to other neurogenic models, is dependent on benzamil blockable mechanisms in brain. The effect of ICV benzamil on AngII‐induced increases in arterial pressure was investigated in rats consuming either a high‐ or low‐salt diet.

## Methods

### Animals and salt diets

Male Sprague‐Dawley rats (250–275 g) were purchased from Charles River Laboratories (Wilmington, MA) and housed in small groups in a temperature‐ and light‐controlled room until the time of study. During this period rats were randomly placed on either a low‐ (0.1% NaCl) or high (2.0% NaCl)‐salt diet (Research Diets, New Brunswick, NJ) with distilled water available ad libitum. Rats remained on these diets for the duration of the study. All procedures were approved by the University of Minnesota Animal Care and Use Committee and were conducted in accordance with the institutional and National Institutes of Health guidelines.

### Instrumentation for cardiovascular measurements and benzamil administration

Rats were anesthetized with an injection of pentobarbital (50 mg/kg, IP), prepared for surgery and given gentamicin (0.05 mL, i.m., Hospira, Lake Forest, IL) for antimicrobial prophylaxis. Surgery was performed using aseptic techniques on a heated pad. Following a midline abdominal incision, a telemetry transmitter (model TA11PA‐C40; Data Sciences; St. Paul, MN) was placed into the abdominal cavity and its catheter was inserted into the abdominal aorta as previously described (Yoshimoto et al. 2010). Rats were then placed in a stereotaxic apparatus (Kopf, Tujunga, CA) for placement of a stainless steel ICV cannula (Alzet BIK2, 3–5 mm; Durect Corp., Cupertino, CA) in the right lateral ventricle using the following coordinates relative to bregma; −0.15 mm posterior, 0.15 mm lateral, and 0.35 mm ventral to the pial surface. A polyethylene catheter was connected to the cannula. Two jewelers' screws were placed in the skull and the catheter was secured to the cannula and skull surface using dental cement (Stoelting Co., Woodale, IL). The distal end of the catheter was tunneled subcutaneously to the scapulae and connected to an Alzet osmotic minipump (model 2004; Durect Corp) for administration of either benzamil or its vehicle at a rate of 0.5 *μ*L/h. Benzamil was initially dissolved in propylene glycol and then diluted with 0.9% saline. The pH of the final solution was adjusted to 7.4 with NaOH. All skin incisions were closed and rats were treated with antibiotics (ampicillin 0.15 mg/kg s.c., Sandoz International, GmbH, Holzkirchen, Germany; tobramycin 3 mg/kg sid, s.c., Teva Pharmaceuticals USA, Irvine, CA) and an analgesic (buprenorphine 0.05 mg/kg, s.c., Reckitt Benckiser Pharmaceuticals, Inc., Richmond, VA). Upon recovery from anesthesia, rats were individually housed in metabolic cages with the telemetry receiver mounted adjacent to them for continuous monitoring of arterial pressure and heart rate. Rats were allowed 5 days for surgical recovery before initiating the experimental protocols.

## Experimental Protocols

Intracerebroventricular benzamil or vehicle was infused continuously during the 5‐day surgical recovery period and experimental protocol. In all protocols, arterial pressure and heart rate were monitored over a period of 17 days: 4 days of baseline followed by 13 days of AngII administration. Upon completion of the 4th baseline day, rats were anesthetized with isoflurane, weighed and then an Alzet osmotic minipump (model 2ML2) filled with AngII was implanted as previously described (Yoshimoto et al. 2010). The concentration of AngII was adjusted to deliver an initial dose of 150 ng/kg/min based on body weight at the time of implantation. Rats were returned to their cage and monitored for 13 days of AngII infusion.

### Protocol 1: effect of ICV administration of 16 nmol/day benzamil on the arterial pressure response to AngII in rats on a high‐salt diet

Two groups of rats were studied in metabolic cages to measure daily sodium and water intake and excretion. The first group (*N* = 8) was treated with ICV benzamil at a dose of 16 nmol/day. The second group (*N* = 10) was treated with ICV vehicle. Twenty‐four‐hour food and water intake and urine output were measured gravimetrically and daily urine samples were taken for analysis of sodium concentration using a NOVA‐5^+^ sodium‐potassium analyzer (Biomedical, Waltham, MA). Twenty‐four‐hour sodium intake was calculated by multiplying the food intake by sodium content of food (2.0% = 0.35 mmol Na/g). Twenty‐four‐hour urinary sodium excretion was calculated as the product of urine flow and urine sodium concentration.

### Protocol 2: effect of ICV administration of 8 nmol/day benzamil on the arterial pressure response to AngII in rats on a high‐salt diet

The objective of this protocol was to determine whether a lower dose of benzamil than that used in Protocol 1 was effective in attenuating AngII–salt hypertension. The protocol was identical to Protocol 1 with the exception that benzamil was administered at a dose of 8 nmol/day. Sodium and water intake and excretion were measured in both groups; ICV benzamil (*N* = 8) and ICV vehicle (*N* = 10).

### Protocol 3: effect of ICV administration of 16 nmol/day benzamil on the arterial pressure response to AngII in rats on a low‐salt diet

The objective of this protocol was to test the hypothesis that the effect of the higher dose of ICV benzamil on arterial pressure in rats administered AngII were dependent on rats consuming a high‐salt diet. This protocol was identical to Protocol 1 with the exception that rats consumed a low‐salt diet (0.1% = 0.018 mmol Na/g). Two groups were studied; ICV benzamil (*N* = 6) and ICV vehicle (*N* = 6). Sodium and water intake and excretion were measured in both groups.

### Data and statistical analysis

Mean arterial pressure (MAP) and heart rate data were sampled over a 10‐sec period at 500 Hz every 4 min and stored for later analysis. Twenty‐four‐hour averages of MAP and heart rate, as well as sodium and water intake and excretion were plotted and analyzed by two‐way analysis of variance for repeated measures followed by the Holm–Sidak method for all post hoc comparisons (SigmaStat version 3.5, San Jose, CA). Statistical significance was set at *P* < 0.05.

## Results

### Effect of ICV administration of benzamil on responses to AngII in rats on a high‐salt diet

**Figure 1.**
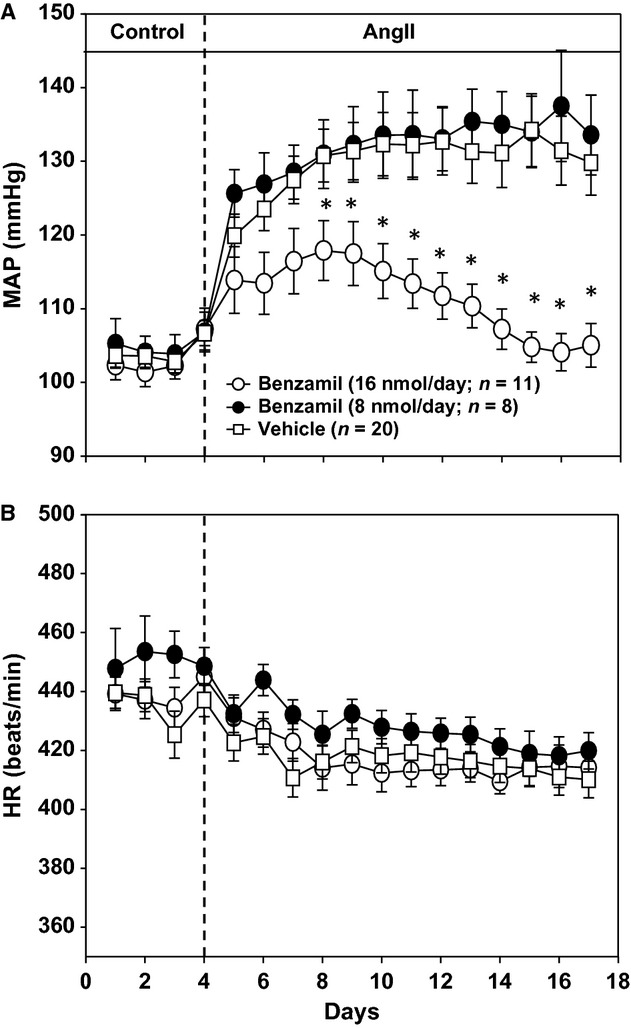
(A) Mean arterial pressure (MAP) response to angiotensin II (AngII) administration in rats consuming a high‐salt diet (2.0% NaCl). Rats were treated with intracerebroventricular (ICV) benzamil at 16 nmol/day (*N* = 11), 8 nmol/day (*N* = 8) or ICV vehicle (*N* = 20). (B) Heart rate (HR) responses in same groups as panel A. There were no statistical differences in HR between groups. *P < 0.05 compared to ICV vehicle group on given day.

**Figure 2.**
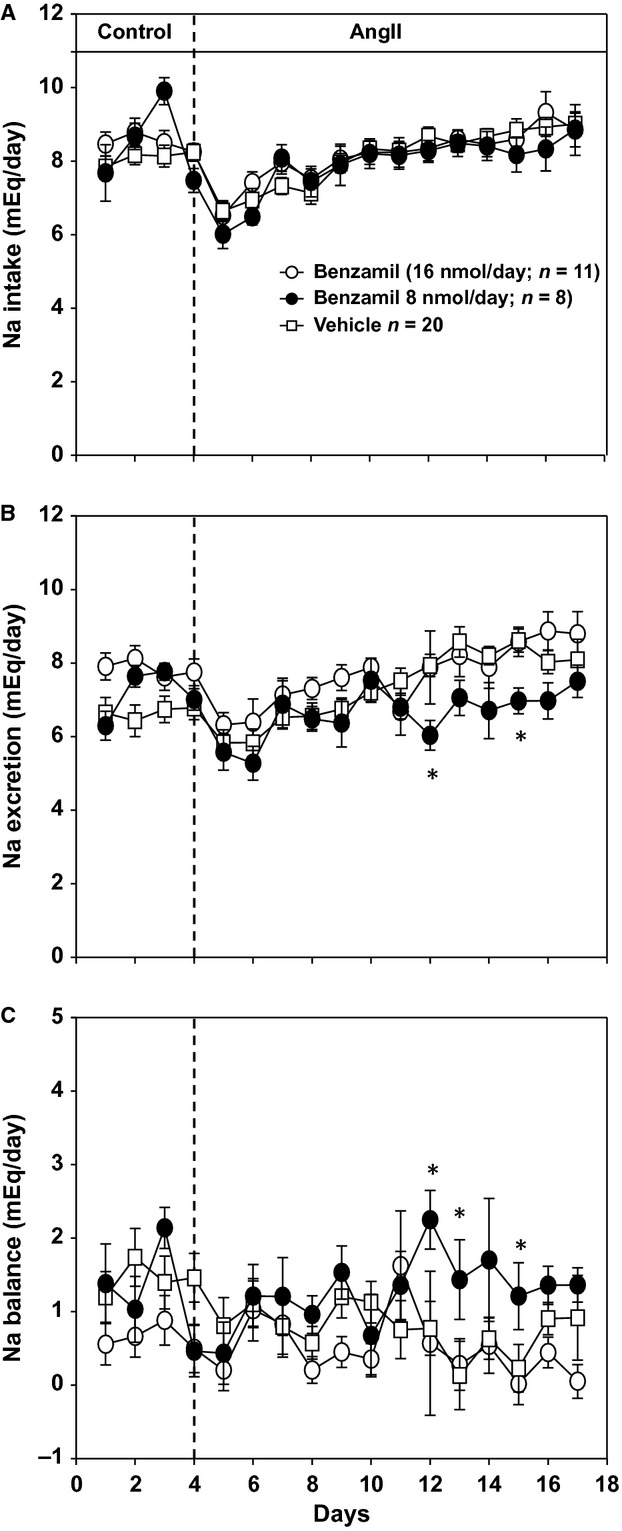
(A) Sodium intake responses to angiotensin II (AngII) administration in rats consuming a high‐salt diet (2.0% NaCl). Treatment groups are the same as in Fig. 1. (B) Sodium excretion responses for same groups as in panel A. (C) Twenty‐four‐hour sodium balance responses for same groups as panels A and B. *P < 0.05 compared to ICV vehicle group on given day.

**Figure 3.**
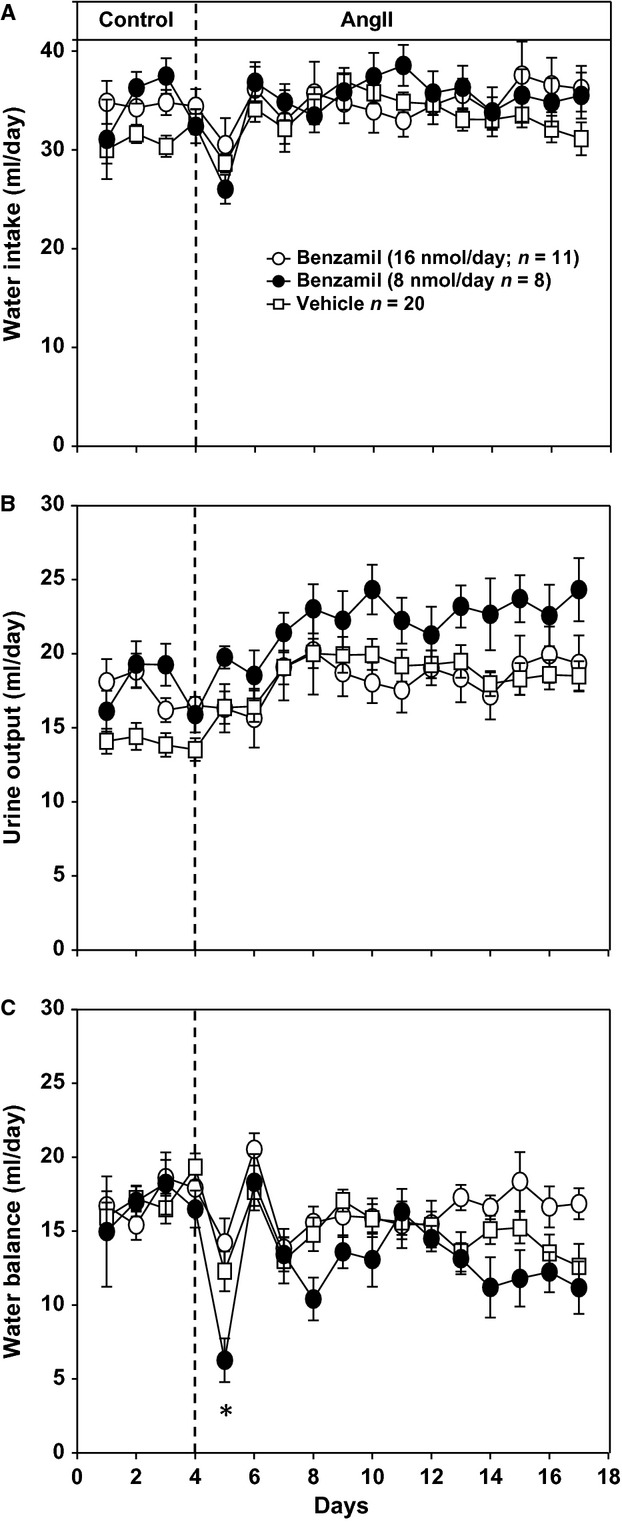
(A) Water intake responses to angiotensin II (AngII) administration in rats consuming a high‐salt diet (2.0% NaCl). Treatment groups are the same as in Fig. 1. (B) Urine output responses for same groups as in Panel A. (C) Twenty‐four‐hour water balance responses for same groups as panels A and B. *P < 0.05 compared to ICV vehicle group on given day.

Two separate protocols were conducted to investigate the effects of two doses of benzamil, 8 and 16 nmol/day, on cardiovascular and fluid balance responses to AngII in rats consuming a high‐salt diet. Each protocol included a vehicle control group. Since responses in vehicle‐treated rats were not statistically different between protocols, data from these groups were combined and are presented with both benzamil groups in Figs. 1–3.

Prior to AngII administration, control levels of MAP were similar in all three groups (Fig. 1A). MAP increased approximately 30 mmHg over the 13‐day period of AngII administration in ICV vehicle‐treated rats and this response was not affected by the lower dose of ICV benzamil (8 nmol/day). In contrast, benzamil at a dose of 16 nmol/day statistically attenuated the MAP response by day 4 of AngII administration and completely prevented the response by the end of the protocol. As shown in Fig. 1B, heart rates were similar in all three groups during the baseline period and throughout the AngII infusion period of the protocol.

Variables for sodium balance are shown in Fig. 2. Compared to the vehicle control group, neither dose of ICV benzamil had an effect on 24‐h sodium intake which averaged ~8 mEq/day throughout the protocol. Since it is not possible to accurately collect 100% of the daily urine output in standard metabolic cages due to evaporative losses off of the funnels, sodium excretion was slightly less than sodium intake, which is reflected as a positive sodium balance. Nonetheless, assuming this measurement error is constant across groups, sodium excretion was similar among all three groups with the exception of the low‐dose benzamil group, which had moderately lower excretions rates on days 12, and 15. As a result sodium balance was also similar between groups with the exception of the low‐dose benzamil group which had higher levels on days 12, 13, and 15 compared to ICV vehicle and high‐dose benzamil groups.

Variables for water balance are shown in Fig. 3. Compared with the vehicle control group, neither dose of ICV benzamil had an effect on 24‐h water intake which averaged ~35 mL/day throughout the protocol. Daily urine output was not statistically different between groups although the low‐dose ICV benzamil group trended toward higher values. Similarly, with the exception of the first day of AngII in which water balance was significantly lower in the low‐dose benzamil group, water balance was similar among all groups.

### Effect of ICV administration of 16 nmol/day benzamil on responses to AngII in rats on a low‐salt diet

**Figure 4.**
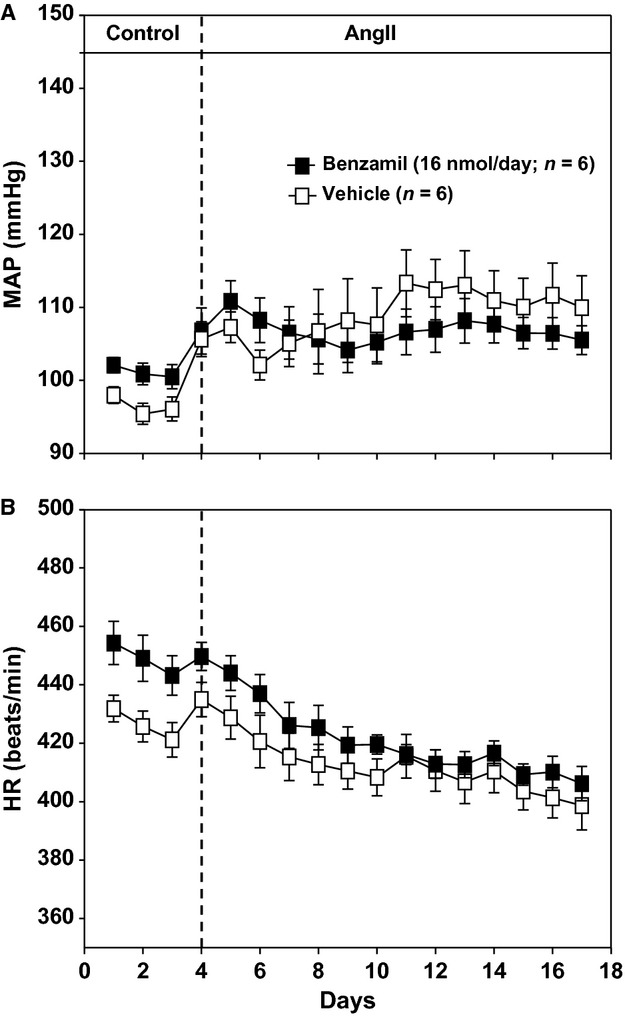
(A) Mean arterial pressure (MAP) response to angiotensin II (AngII) administration in rats consuming a low‐salt diet (0.1% NaCl). Rats were treated with either intracerebroventricular (ICV) benzamil at 16 nmol/day (*N* = 6) or ICV vehicle (*N* = 6). There were no statistical differences within or between treatment groups. (B) Heart rate (HR) responses in same groups as panel A. There were no statistical differences in HR between groups.

This protocol was conducted to determine whether ICV benzamil affected the arterial pressure response to AngII in the absence of a high‐salt diet. As seen in Fig. 4A and reported previously (Osborn and Fink 2010), the MAP response to AngII was minimal in rats consuming a low‐salt diet as represented by the ICV vehicle group. Not surprisingly, there were no differences between the ICV benzamil‐ and vehicle‐treated groups. Similarly, there were no differences in heart rate between these two groups (Fig. 4B).

**Figure 5.**
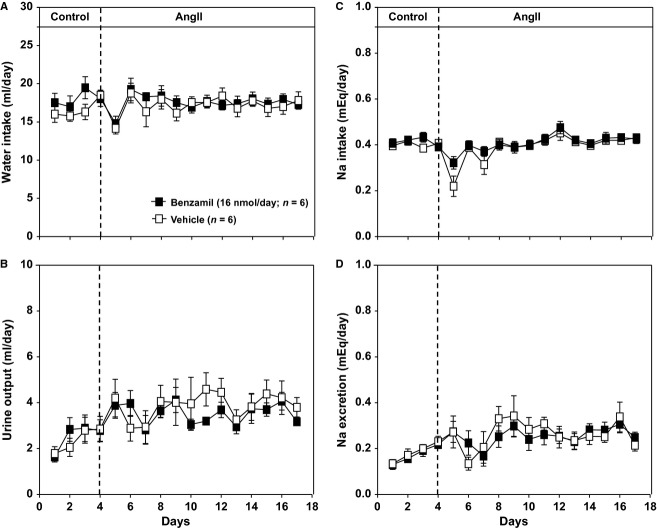
(A) Water intake response to Angiotensin II (AngII) administration in rats consuming a low‐salt diet (0.1% NaCl). Treatment groups are the same as in shown in Fig. 4. There were no statistical differences within or between treatment groups. (B) Urine output response to Angiotensin II (AngII) administration in rats consuming a low‐salt diet (0.1% NaCl). Treatment groups are the same as shown in Fig. 4. There were no statistical differences within or between treatment groups. (C) Sodium (Na) intake response to Angiotensin II (AngII) administration in rats consuming a low‐salt diet (0.1% NaCl). Treatment groups are the same as shown in Fig. 4. There were no statistical differences within or between treatment groups. (D) Sodium (Na) excretion response to Angiotensin II (AngII) administration in rats consuming a low‐salt diet (0.1% NaCl). Treatment groups are the same as shown in Fig. 4. There were no statistical differences within or between treatment groups.

Daily water intake in rats consuming a 0.1% NaCl diet (Fig. 5A) was approximately 50% of that observed in rats on a 2.0% NaCl diet (Fig. 3A). There were no differences between the ICV benzamil‐ and vehicle‐treated groups for water intake or urine output (Fig. 5B). Daily sodium intake in rats consuming a 0.1% NaCl diet was ~0.4 mEq/day (Fig. 5C) in contrast to ~8 mEq/day observed in rats on a 2.0% NaCl diet (Fig. 2A). There were no differences between the ICV benzamil‐ and vehicle‐treated groups for sodium intake or excretion (Fig. 5D). Finally, there were no differences in water and sodium balance (data not shown) between ICV benzamil‐ and ICV vehicle‐treated groups.

## Discussion

This study tested the hypothesis that chronic ICV administration of the sodium channel/transporter blocker benzamil would attenuate or prevent hypertension in the AngII–salt model in the rat. Several previous studies provided support for our hypothesis. First, the contribution of the sympathetic nervous system to AngII‐induced hypertension is amplified by a high‐salt diet (King and Fink 2006, 2008; Osborn and Fink 2010), suggesting an interaction between sodium sensing sites in the brain and circulating AngII. Second, ICV benzamil blocks the pressor (Nishimura et al. 1998; Abrams and Osborn 2008) and sympathoexcitatory response (Nishimura et al. 1998) to an acute increase in cerebrospinal fluid (CSF) sodium concentration. Third, ICV benzamil also prevents hypertension produced by long‐term increases in CSF sodium concentration (Huang and Leenen 2002). Finally, chronic ICV benzamil attenuates or reverses three other “neurogenic salt sensitive” models of hypertension; the stroke‐prone spontaneously hypertensive rat (Nishimura et al. 1998), the Dahl‐S rat (Wang and Leenen 2002), and the DOCA‐salt model (Nishimura et al. 1998; Abrams et al. 2010). In contrast, ICV benzamil does not affect salt‐resistant forms of hypertension that have been tested such as the aortic ligation renovascular model (Nishimura et al. 1998).

Collectively, several studies from our group suggest that the “neurogenic phase” of the AngII–salt hypertension model used in this study does not begin until the 5th day of AngII administration (King and Fink 2008; Osborn et al. 2011; Kuroki et al. 2012). This is consistent with the present findings in which the arterial pressure response to AngII was similar in rats treated with the high dose of ICV benzamil, compared to those treated with ICV vehicle during the initial “nonneurogenic” period. However, arterial pressure subsequently returned to control levels in the ICV benzamil group such that rats were normotensive by the end of the AngII period. By the final day of AngII administration, arterial pressure was approximately 30 mmHg lower in ICV benzamil compared to ICV vehicle‐treated rats. Further support for the hypothesis that benzamil blocks a synergistic interaction between AngII and dietary sodium is that AngII did not increase arterial pressure in rats on a low‐salt diet and, not surprisingly, ICV benzamil had no effect on arterial pressure in AngII‐treated rats consuming a low‐salt diet. We acknowledge that the latter observation is not unequivocal proof that high‐salt intake is required for activation of a benzamil blockable sympathoexcitatory mechanism in AngII‐treated rats, but when failure of benzamil to lower blood pressure in rats consuming a low‐salt diet is considered together with our data showing that it does reduce blood pressure in high‐salt fed rats, it seems highly likely that high‐salt intake and Ang II together are sufficient to recruit a benzamil‐sensitive mechanism that supports neurogenic hypertension. Of note, failure of benzamil to lower blood pressure in AngII‐treated rats consuming a low‐salt diet clearly indicates that the blood pressure lowering effect of benzamil in AngII–salt hypertensive rats is not attributable to a nonspecific sympathoinhibitory effect. Collectively, data from this study are consistent with the conclusion that hypertension caused by AngII administration to rats consuming a high‐salt diet is dependent on “benzamil blockable sodium mobilizing proteins” in the brain.

Although we conclude that ICV benzamil prevented the delayed neurogenic phase of AngII–salt hypertension, we did not measure, directly or indirectly, sympathetic activity in this study. Previous reports from our group suggest increased sympathetic activity is specific to the splanchnic vascular bed (King et al. 2007; Osborn et al. 2011; Kuroki et al. 2012) and that sympathetic discharge to the kidneys and skeletal muscle is decreased or unchanged respectively (Yoshimoto et al. 2010). In addition, changes in cardiac sympathetic activity do not appear to contribute to hypertension in this model since neither chronic administration of the beta‐1 adrenoceptor antagonist, atenolol, or denervation of cardiac sympathetic nerves by stellate ganglionectomy affect the arterial pressure or heart response to AngII in high‐salt rats (Hirsch and Osborn 2011). This is consistent with this study in which there were no effects of ICV benzamil on the heart rate response to AngII administration in high‐salt rats. Therefore, it is uncertain whether indirect markers of global sympathetic activity, such as urinary catecholamine metabolites, would have been affected by ICV benzamil. Additional studies are needed in which splanchnic nerve activity is directly recorded before and during AngII administration in control and ICV benzamil‐treated rats. Although this approach is technically challenging, it is the most direct test of our hypothesis.

Beyond providing additional evidence that benzamil blockable proteins in the brain contribute to another model of neurogenic salt‐sensitive hypertension, this study indicates that these channels/transporters are likely to participate in sympathetic regulation of blood pressure under more physiological salt‐loading conditions than has previously been recognized. In this study, salt loading was achieved by providing 2.0% NaCl diet ad libitum which resulted in a daily sodium intake of ~8 mmol/day. The rats had ad libitum access to water during the entire protocol. This salt‐loading paradigm does not result in a significant increase in plasma osmolality (unpublished observations). In contrast, salt loading in the DOCA‐salt model is achieved by giving rats 0.9% saline to drink, without access to water, which results in a 24‐h sodium intake of ~20 mmol/day (Jacob et al. 2005) and significant increases in plasma osmolality (Veitenheimer and Osborn 2011). A similar method of salt loading (i.e., saline drinking solution) was used in a study of stroke‐prone spontaneously hypertensive rats‐ in which ICV benzamil lowered arterial pressure (Nishimura et al. 1998). Similarly‐ high levels of daily sodium intake have been used to study the role of ICV benzamil in the Dahl‐S rats which are given a salt diet that contains a fourfold higher sodium chloride content (8.0% NaCl; Gomez‐Sanchez and Gomez‐Sanchez 1995; Huang and Leenen 2002) than used in this study. Although the physiological relevance of the effects of ICV benzamil may be questioned in these earlier studies, due to the supraphysiological levels of salt loading used, the current study provides new evidence that similar mechanisms may play a role in the effects of ICV benzamil in the more physiological salt‐loading paradigm employed in the AngII–salt model. This is clinically relevant in regards to the possible role of brain sodium dysregulation in salt‐sensitive forms of human hypertension and development of new therapeutic strategies.

Precisely which molecular target(s) of benzamil may account for its efficacy in this study is unknown as is its location in brain autonomic pathways. Ion channels permeable to sodium, such as ENaC or acid‐sensitive ion channels (ASIC) are likely candidates (Huang et al. 2006; Abrams and Osborn 2008). Both ENaC and ASIC are members of the Degenerin/Epithelial Sodium Channel (Deg/ENaC) superfamily of ion channels. ENaC have long been known for their role in promoting sodium transport from urine across principal cells of renal tubules, and are thought to play a critical role in maintaining normal arterial pressure (Lele 2004; Bhalla and Hallows 2008). In addition, both ENaC and ASIC may play a broad role in maintaining pressure, including in local pressure‐induced vasoconstriction and in modulating the baroreceptor reflex (Drummond et al. 2008). Another large group of benzamil‐sensitive proteins include ion transport systems, such as the Na^+^/H^+^ exchanger, the Na^+^/Ca^++^ exchanger, the Na^+^/K^+^ ATPase, and the Ca^++^ pump (Abrams and Osborn 2008). These proteins may play a role in the development of certain forms of hypertension; the Na^+^/H^+^ exchanger, for instance, has been implicated in the development of hypertension in humans (Siffert and Dusing 1995). However, while amiloride and benzamil are inhibitors of these ion transport systems, these proteins have higher affinity for other amiloride analogues, such as 3′,4′‐dichlorobenzamil; 2′,4′‐dimethylbenzamil; 5‐(N‐ethyl‐N‐isopropyl) amiloride; and 5‐(N‐methyl‐N‐isobutyl) amiloride (Keep et al. 1999). Benzamil is only significantly higher than what is necessary to inactivate ENaC or ASIC.

We did not measure CSF concentration of benzamil in this study, but it is possible to make a rough estimate. Although the total volume of CSF in the rat varies with size, a reasonable estimate for rats used in this study is 300 *μ*l (Bass and Lundborg 1973). Given delivery of 16 nmol of benzamil over a 24‐h time period, it is estimated that CSF benzamil concentration would increase by ~50 *μ*mol/L per day – assuming no clearance/degradation of benzamil or turnover of CSF. However, rats produce ~5300 *μ*L of CSF over a 24‐h period (Harnish and Samuel 1988). Accordingly, the CSF volume is replaced approximately every 1.5 h, or ~16 times per day. Thus, a more accurate estimate of steady‐state CSF benzamil concentration is in the low nanomolar range (3–5 nmol/L). This is below the IC_50_ for ENaC subunits, ASIC subunits and ion exchangers, including the Na/Ca exchanger, which has the highest affinity for benzamil among the exchangers (Keep et al. 1999). Therefore, we suggest that effects of ICV benzamil observed in this study are likely due to actions at proteins localized to brain regions in the vicinity of the tip of the ICV cannula.

Although we observed that ICV benzamil at a dose of 16 nmol/day abolished the neurogenic phase of AngII–salt hypertension, 8 nmol/day had no effect. It is somewhat surprising that a twofold difference in dose made the difference between complete reversal of hypertension and no effect. As suggested above, this may be related to positioning of the tip of the ICV cannula and the site of action. It should be noted that a wide range of doses of ICV benzamil has been reported by others to attenuate or block models of salt‐sensitive hypertension. Nishimura and colleagues reported that a lower dose than that used in our study, 3 nmol/day, attenuated mineralocorticoid hypertension (Nishimura et al. 1998). On the other hand, much higher doses have been administered in studies using the Dahl salt‐sensitive rat. Gomez‐Sanchez showed that 75 nmol/day blocked hypertension in this model (Gomez‐Sanchez and Gomez‐Sanchez 1995), whereas another group reported similar results using 90 nmol/day (Wang and Leenen 2002). Direct comparison of effective doses between studies is complicated by the use of different experimental models and possible differences in the position of the infusion ICV cannula. The method of measurement for arterial pressure is also likely to complicate comparison between studies. Methods range from acute indirect tail cuff measurement in restrained rats (Gomez‐Sanchez and Gomez‐Sanchez 1995; Nishimura et al. 1998), to acute direct measurement 1 day following catheter implantation (Wang and Leenen 2002), to chronic continuous telemetric measurement in unrestrained rats in the present investigation.

Proposed sites of action of benzamil include sodium‐sensitive structures in the lamina terminalis such as the subfornical organ (SFO) and organum vasculosum of the lamina terminalis (OVLT), which process and relay information downstream to sympathetic control neurons in the paraventricular nucleus of the hypothalamus (PVN) and rostral ventrolateral medulla (RVLM). We have recently reported that whereas electrolytic lesion of the SFO has minimal effects (Osborn et al. 2012),OVLT lesion markedly attenuates AngII–salt hypertension in rats (Collister et al. 2013). The area that has received the most attention in this regard is the PVN (Wang et al. 2010). We recently reported that the same dose of ICV benzamil used in this study (16 nmol/day) attenuated both the rise of arterial pressure and chronic activation of PVN neurons of DOCA‐salt hypertensive rats (Abrams et al. 2010). Another group reported the presence of mRNA for all three ENaC subunits in PVN samples (3). However, all three subunits have yet to be localized to individual neurons (Amin et al. 2005) and thus it remains to be proven that functional ENaC exist in, and are capable of modulating the activity of, PVN neurons. To whatever extent ENaC expressed in PVN might be capable of inducing or modulating neuronal discharge, it appears from available evidence that constitutive ENaC activity in the PVN of normotensive rats does not modulate neuronal activity. This is the case because direct PVN microinjection of hypertonic NaCl‐based Ringer's solution does not acutely alter blood pressure or lumbar SNA (Atunes et al. 2006). This notwithstanding, it remains possible that upregulation of ENaC expression could occur in the PVN of AngII–salt hypertensive rats and contribute to activation SNA and elevation of arterial pressure. Additional experiments are needed to test this hypothesis.

Another possibility is that benzamil administered ICV does not act on neurons, or glia, but affects sodium transport between blood and cerebrospinal fluid (CSF) to ultimately affect CSF sodium concentration. This would secondarily affect sodium‐sensitive neurons that subsequently drive sympathetic activity. Since benzamil has a low molecular weight (356.2 Da) and is lipophilic relative to other amiloride analogs (Hofmann et al. 1998), it is possible that benzamil in the CSF exits through intercellular “gaps” between cerebral ventricular choroid plexus epithelial cells and inhibits a Na^+^ conductive pathway located in the basolateral membrane (blood side). This would decrease CSF sodium concentration and therefore activity of osmotically driven sympathetic activity (Toney and Stocker 2010). Functional evidence for the existence of benzamil‐sensitive Na^+^ channels in choroid plexus cells is lacking, although ENaC subunits have been detected in the basolateral membrane by immunohistochemistry (1). It has been proposed that ENaC in the choroid plexus control the level of sodium in hypothalamic tissue independent from other brain sites (Wang et al. 2010) but this hypothesis has not been confirmed (Praetorius 2007). A recent study suggests that ICV benzamil decreases hypothalamic sodium content by blockade of ENaC in the choroid plexus (Wang et al. 2010). In that study, it was concluded that ENaC in neural sites such as the subfornical organ and PVN were not a factor in neurogenic salt‐sensitive hypertension.

In summary, this study suggests that the sodium‐sensitive neurogenic component of AngII‐induced hypertension is dependent on sodium channels and/or transporters in the brain that are blocked by benzamil. The results of this study, combined with those in other models of neurogenic salt‐sensitive hypertension, are consistent with an emerging unifying hypothesis that sympathetic nervous system activation significantly contributes to salt‐sensitive hypertension. Future studies directed toward elucidating the molecular target(s) and brain site(s) at which benzamil acts are needed to possibly translate these findings into new therapies for the treatment of salt‐sensitive hypertension.

## Acknowledgments

The authors would thank Myraida Rodriquez for her technical assistance with these studies.

## Conflict of Interest

None declared.

## References

[b1] Abrams J. M. Osborn J. W. 2008 A role for benzamil‐sensitive proteins of the central nervous system in the pathogenesis of salt‐dependent hypertension. Clin. Exp. Pharmacol. Physiol.; 35:687-694 18387084 10.1111/j.1440-1681.2008.04929.x PMC2693203

[b2] Abrams J. M. Engeland W. C. Osborn J. W. 2010 Effect of intracerebroventricular benzamil on cardiovascular and central autonomic responses to DOCA‐salt treatment. Am. J. Physiol.; 299:R1500-R1510 10.1152/ajpregu.00431.2010 PMC3007181 20926762

[b3] Amin M. S. Wang H. W. Reza E. Whitman S. C. Tuana B. S. Leenen F. H. 2005 Distribution of epithelial sodium channels and mineralocorticoid receptors in cardiovascular regulatory centers in rat brain. Am. J. Physiol.; 289:R1787-R1797 10.1152/ajpregu.00063.2005 16141309

[b4] Atunes V. R. Yao S. T. Pickering A. E. Murphy D. Paton J. F. R. 2006 A spinal vasopressinergic mechanism mediates hyperosmolality‐induced sympathoexciation. J. Physiol.; 576:569-583 16873404 10.1113/jphysiol.2006.115766 PMC1890358

[b5] Bass H. N. Lundborg P. 1973 Postnatal development of bulk flow in the cerebrospinal fluid of the albino rat: clearance of carboxyl‐[14c]inulin after intrathecal infusion. Brain Res.; 52:323-332 4739806 10.1016/0006-8993(73)90668-9

[b6] Bhalla V. Hallows K. R. 2008 Mechanisms of regulation and clinical implications. J. Am. Soc. Nephrol.; 19:1845-1854 18753254 10.1681/ASN.2008020225

[b7] Collister J. P. Olson M. K. Nahey D. B. Vieira A. A. Osborn J. W. 2013 Ovlt lesion decreases basal arterial pressure and the chronic hypertensive response to AngII in rats on a high‐salt diet. Physiol. Rep.; 1:1-9 10.1002/phy2.128 PMC3841056 24303192

[b8] Cowley A. W. Jr McCaa R. E. 1976 Acute and chronic dose‐response relationships for angiotensin, aldosterone, and arterial pressure at varying levels of sodium intake. Circ. Res.; 39:788-797 1000772 10.1161/01.res.39.6.788

[b9] Drummond H. A. Jernigan N. L. Grifoni S. C. 2008 Sensing tension: epithelial sodium channel/acid‐sensing ion channel proteins in cardiovascular homeostasis. Hypertension; 51:1265-1271 18378856 10.1161/HYPERTENSIONAHA.107.093401 PMC2788303

[b10] Gomez‐Sanchez E. P. Gomez‐Sanchez C. E. 1995 Effect of central infusion of benzamil on Dahl S rat hypertension. Am. J. Physiol.; 269:H1044-H1047 7573500 10.1152/ajpheart.1995.269.3.H1044

[b11] Harnish P. P. Samuel K. Reduced cerebrospinal fluid production in the rat and rabbit by diatrizoate. Ventriculocisternal perfusion. Invest. Radiol. 1988; 23:534-536 3170143 10.1097/00004424-198807000-00010

[b12] Hirsch D. M. Osborn J. Cardiac sympathetic nerves do not contribute to AngII‐salt hypertension. FASEB J. 2011; 25:640.8

[b13] Hofmann T. Stutts M. J. Ziersch A. Ruckes C. Weber W. M. Knowles M. R. 1998 Effects of topically delivered benzamil and amiloride and nasal potential difference in cystic fibrosis. Am. J. Respir. Crit. Care Med.; 157:1844-1849 9620916 10.1164/ajrccm.157.6.9709043

[b14] Huang B. S. Leenen F. H. Brain amiloride‐sensitive phe‐met‐arg‐phe‐nh2‐gated na channels and Na‐induced sympathoexcitation and hypertension. Hypertension. 2002; 39part 2:557-561 11882607 10.1161/hy02t2.103004

[b15] Huang B. S. Amin M. S. Leenen F. H. 2006 The central role of the brain in salt‐sensitive hypertension. Curr. Opin. Cardiol.; 21:295-304 16755197 10.1097/01.hco.0000231398.64362.94

[b16] Jacob F. Clark L. A. Guzman P. A. Osborn J. W. 2005 Role of renal nerves in development of hypertension in DOCA‐salt model in rats: a telemetric approach. Am. J. Physiol.; 289:H1519-H1528 10.1152/ajpheart.00206.2005 15937098

[b17] Keep R. F. Si X. Shakui P. Ennis S. R. Betz A. L. 1999 Effect of amiloride analogs on DOCA‐salt‐induced hypertension in rats. Am. J. Physiol.; 276:H2215-H2220 10362706 10.1152/ajpheart.1999.276.6.H2215

[b18] King A. J. Fink G. D. 2006 Chronic low‐dose angiotensin II infusion increases venomotor tone by neurogenic mechanisms. Hypertension; 48:927-933 17000931 10.1161/01.HYP.0000243799.84573.f8

[b19] King A. J. Fink G. D. 2008 Whole body norepinephrine kinetics in AngII‐salt hypertension in the rat. Am. J. Physiol. (Reg. Int. Comp).; 294:R1262-R1267 10.1152/ajpregu.00819.2007 18256139

[b20] King A. J. Osborn J. W. Fink G. D. 2007 Splanchnic circulation is a critical neural target in angiotensin II salt hypertension in rats. Hypertension; 50:547-556 17646575 10.1161/HYPERTENSIONAHA.107.090696

[b21] Kuroki M. T. Guzman P. A. Fink G. D. Osborn JW 2012 Time dependent changes in autonomic control of splanchnic vascular resistance and heart rate in AngII‐salt hypertension. Am. J. Physiol.; 302:H763-H769 10.1152/ajpheart.00930.2011 PMC3353774 22114134

[b22] Lele R. D. 2004 Hypertension: molecular approach. J. Assoc. Physicians India; 52:53-62 15633721

[b23] Nishimura M. Ohtsuka K. Nanbu A. Takahashi H. Yoshimura M. 1998 Benzamil blockade of brain Na channels averts Na‐induced hypertension in rats. Am. J. Physiol.; 274:R635-R644 9530228 10.1152/ajpregu.1998.274.3.R635

[b24] Osborn J. W. Fink G. D. 2010 Region specific changes in sympathetic nerve activity in AngII‐salt hypertension. Exp. Physiol.; 95:61-68 19717492 10.1113/expphysiol.2008.046326 PMC2856071

[b25] Osborn J. W. Fink G. D. Kuroki M. T. 2011 Neural mechanisms of angiotensin II‐salt hypertension: implications for therapies targeting neural control of the splanchinc circulation. Curr. Hypertens. Rep.; 13:221-228 21298369 10.1007/s11906-011-0188-9 PMC3076522

[b26] Osborn J. W. Hendel M. Collister J. P. Fink G. D. 2012 Role of the subfornical organ in angiotensin II‐salt hypertension. Exp. Physiol.; 97:80-88 21967900 10.1113/expphysiol.2011.060491 PMC3253211

[b27] Peti‐Peterdi J. Warnock D. G. Bell P. D. 2002 Angiotensin II directly stimulates ENaC activity in the coritical collecting duct via AT1 receptors. J. Am. Soc. Nephrol.; 13:1131-1135 11960999 10.1097/01.asn.0000013292.78621.fd

[b28] Praetorius J. 2007 Water and solute secretion by the choroid plexus. Pflugers Arch. Eur. J. Physiol.; 454:1-18 17120021 10.1007/s00424-006-0170-6

[b29] Siffert W. Dusing R. 1995 Sodium‐proton exchange and primary hypertension. An update. Hypertension; 26:649-655 7558226 10.1161/01.hyp.26.4.649

[b30] Toney G. M. Stocker S. D. 2010 Hyperosmotic activation of cns sympathetic drive: implications for cardiovascular disease. J. Physiol.; 588:3375-3384 20603334 10.1113/jphysiol.2010.191940 PMC2988504

[b31] Veitenheimer B. Osborn J. W. 2011 Role of spinal V1a receptors in regulation of arterial pressure during acute and chronic osmotic stress. Am. J. Physiol.; 300:R460-R469 10.1152/ajpregu.00371.2010 PMC3043806 21123759

[b32] Wang H. Leenen F. H. 2002 Brain sodium channels mediate increases in brain “ouabain” and blood pressure in Dahl S rats. Hypertension; 40:96-100 12105145 10.1161/01.hyp.0000022659.17774.e4

[b33] Wang H. W. Amin M. S. El‐Shahat E. Huang B. S. Tuana B. S. Leenen F. H. Effects of central sodium on epithelial sodium channels in rat brain. Am. J. Physiol. (Reg. Int. Comp). 2010; 299:R222-R233 10.1152/ajpregu.00834.2009 20427723

[b34] Yoshimoto M. Miki K. Fink G. D. King A. Osborn J. W. 2010 Chronic angiotensin II infusion causes differential responses in regional sympathetic nerve activity in rats. Hypertension; 55:644-651 20100996 10.1161/HYPERTENSIONAHA.109.145110 PMC2856065

